# A generic approach for the design of whole-genome oligoarrays, validated for genomotyping, deletion mapping and gene expression analysis on *Staphylococcus aureus*

**DOI:** 10.1186/1471-2164-6-95

**Published:** 2005-06-17

**Authors:** Yvan Charbonnier, Brian Gettler, Patrice François, Manuela Bento, Adriana Renzoni, Pierre Vaudaux, Werner Schlegel, Jacques Schrenzel

**Affiliations:** 1 Genomic Research Laboratory, University Hospitals of Geneva, rue Micheli-du-Crest 24, CH-1211 Geneva 14, Switzerland; 2 Fondation pour Recherches Médicales, University of Geneva, avenue Roseraie 64, CH-1211 Geneva 4, Switzerland; 3 Service of Infection Diseases, University Hospitals of Geneva, rue Micheli-du-Crest 24, CH-1211 Geneva 14, Switzerland; 4 Clinical Microbiology Laboratory, University Hospitals of Geneva, rue Micheli-du-Crest 24, CH-1211 Geneva 14, Switzerland

## Abstract

**Background:**

DNA microarray technology is widely used to determine the expression levels of thousands of genes in a single experiment, for a broad range of organisms. Optimal design of immobilized nucleic acids has a direct impact on the reliability of microarray results. However, despite small genome size and complexity, prokaryotic organisms are not frequently studied to validate selected bioinformatics approaches. Relying on parameters shown to affect the hybridization of nucleic acids, we designed freely available software and validated experimentally its performance on the bacterial pathogen *Staphylococcus aureus*.

**Results:**

We describe an efficient procedure for selecting 40–60 mer oligonucleotide probes combining optimal thermodynamic properties with high target specificity, suitable for genomic studies of microbial species. The algorithm for filtering probes from extensive oligonucleotides libraries fitting standard thermodynamic criteria includes positional information of predicted target-probe binding regions. This algorithm efficiently selected probes recognizing homologous gene targets across three different sequenced genomes of *Staphylococcus aureus*. BLAST analysis of the final selection of 5,427 probes yielded >97%, 93%, and 81% of *Staphylococcus aureus *genome coverage in strains N315, Mu50, and COL, respectively. A manufactured oligoarray including a subset of control *Escherichia coli *probes was validated for applications in the fields of comparative genomics and molecular epidemiology, mapping of deletion mutations and transcription profiling.

**Conclusion:**

This generic chip-design process merging sequence information from several related genomes improves genome coverage even in conserved regions.

## Background

Current hybridization technologies allow assaying thousands of nucleic acid sequences in a single reaction on a solid substrate. Such massively parallel systems offer unprecedented opportunities for basic research and diagnostic applications, including gene sequencing [1], detection of genetic polymorphisms [2], genome-composition analysis [3,4] and measurement of gene expression profiles in prokaryotes [5,6] or cancer cells [7]. Oligonucleotide probes (up to 70-mer) offer more flexibility than cDNA probes since they can be tailored according to optimal *in silico *physico-chemical and specificity properties, and applied to any sequence data.

Early available probe design software identified sets of probes sharing homogeneous thermodynamic properties for probe-target hybridization [8]. More elaborated software tools include cross-homology testing of probes against a reference database by BLAST (Basic Local Alignment Search Tool) [9,10] or prediction of secondary structures into the thermodynamically-based approach  [11-14]. A frequent drawback of some of these algorithms is to yield an excessive number of unprocessed BLAST outputs that complicates final selection of the most specific probes. Furthermore, these approaches do not take into consideration probe interaction with microarray surface, in particular the impact of mismatches position between the target and probes, as shown by Hughes *et al *[15]. Designing reliable oligonucleotide probes with available software is quite difficult for bacterial genomes with low GC content [16], low complexity in sequence composition, or frequent conserved repeats leading to erroneous target identification by cross-hybridization.

The reported method (OliCheck) implements an algorithm for filtering oligonucleotide probes libraries sharing homogeneous thermodynamic properties by using positional information of predicted target-probe binding regions. An additional characteristic of OliCheck is to annotate probes recognizing highly conserved targets shared by different genomes. *Staphylococcus aureus *(*S. aureus*) was selected as a model organism for implementing and experimentally validating this approach. The choice of this clinically important pathogen for fundamental and applied genomic studies is prompted by the availability of several fully or partially sequenced strain genomes [16-18]. A set of feature elements was designed by OliCheck to yield an extensive *S. aureus *genome coverage. This *S. aureus *specific probe set together with control probes were used to manufacture an oligoarray that was extensively validated for comparative genomics, molecular epidemiology, mapping of deletion mutations, and transcription profiling applications. The specificity, signal-response linearity, and influence of hybridization temperatures for transcript profiling are also described. Further genomic oligoarrays of several distinct microbial species have been successfully designed using this generic methodological approach.

## Results

### Design of a *S. aureus *oligoarray

**Figure 1:**
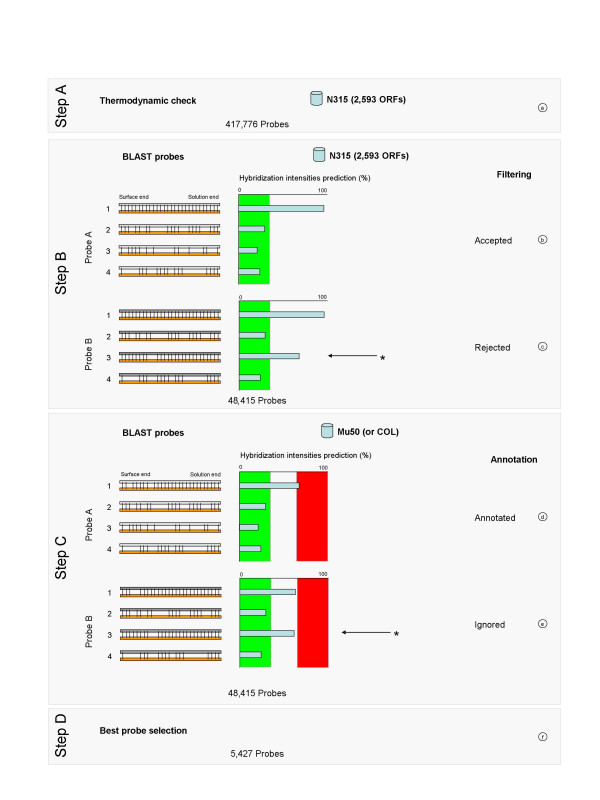
**Schematic representation of StaphChip probe selection**. All ORFs of N315 were loaded into ArrayDesigner™ (a) to select oligonucleotides according to their thermodynamical properties (Step A). The 417,776 N315-derived probes were filtered for target specificity using BLAST against N315 genome (Step B). Each probe should recognize a single target yielding a defined signal intensity threshold, i.e. outside the green box (b), otherwise it is rejected (c). During Step C, all accepted probes are aligned against heterologous *S. aureus *genomes (i.e. Mu50 or COL) to annotate probes common to the other genomes (d, e). Each probe should recognize a single target yielding a defined signal intensity threshold, i.e. inside the red box (d), with no other signals outside the green box; otherwise it is ignored (e). The process is repeated from Step A to C with the two other strain databases. Final selection by spreadsheet analysis (Step D) yielded a total number of 5,427 probes hybridizing with one or more *S. aureus *genomes.

The major genomic component of *S. aureus *is a 2.8 million base pairs (bp) circular chromosome with a low GC content (32.8%) which represents 2,595 ORFs in strain N315. The median length of strain N315 ORFs is 768 bp and the 25^th^–75^th ^percentile ranges from 444-1,152 bp [16]. A probe design software (i.e. ArrayDesigner™) generated a comprehensive list of candidate 40–60 mer probes for each ORF of strain N315 according to the preset thermodynamic hybridization parameters (Fig. 1, step A). A similar process was performed with strain Mu50 and COL. This step yielded 417,776, 607,461 and 321,286 candidate probes for strains N315, Mu50 and COL, respectively.

Further selection of candidate oligonucleotides to sort out cross-reactive probes within the genome of each strain was achieved by OliCheck. This specificity filtering test is a mathematical transposition of Hughes *et al *observations [15] on the impact of mismatches position between the target and probe with respect to the microarray surface. The occurrence of mismatch(es) in the distal portion (solution end) of the probe leads to a strong decrease in signal intensity, as opposed to the proximal portion (surface end). The results of the specificity filtering test, when performed separately on each strain, yielded a list of 48,415, 48,510, and 34,303 probes for N315, Mu50 and COL, respectively (Fig. 1, step B). In contrast to OliCheck-filtered probes that are expected to be devoid of cross-hybridization, >85% of the probes selected by ArrayDesigner™ alone displayed significant cross-hybridization with multiple ORFs.

For each strain, all accepted probes were further annotated by OliCheck against heterologous *S. aureus *genomes to identify probes common to the different genomes (Fig. 1, step C).

To fulfill the manufacturing requirements of a *S. aureus *genome-wide oligoarray, a further probe selection was performed. This selection used a spreadsheet program to rank probes according to optimal strain coverage and thermodynamic criteria, for providing one or more non-overlapping probes per target ORF (Fig. 1, step D).

### *In silico *properties of the *S. aureus *oligoarray and manufacturing of StaphChip

The final set of 5,335 *S. aureus *OliCheck-filtered probes recognized 97.5, 93.0, and 81.0% of N315, Mu50, and COL ORFs, respectively. The low residual percentage of ORFs (2.5% for strain N315 and 7.0% for Mu50) that escaped recognition by our final probe set mostly included (51/65 ORFs for N315) mobile genetic elements, located either on prophage or transposon elements. A likely explanation for exclusion of these targets by OliCheck is the occurrence of highly homologous (>98% nucleotide identity) sequence repeats found in other ORFs. Accordingly, 92 residual probes covering these ORFs had to be selected relying on step A (Fig. 1) only.

To manufacture StaphChip, a total of 5,427 *S. aureus *probes were synthesized on the array together with a subset of control probes designed by OliCheck against *E. coli *K12 genome [19] (see methods).

### Comparison of OliCheck with a currently used method

To validate the properties of OliCheck by comparison with an established method, we generated 60-mer oligonucleotides probes with homogenous thermodynamic criteria for N315 genome by the recently published ArrayOligoSelector [13] tool. The final set of probes generated according to the ArrayOligoSelector procedure [13] (n = 2,592) was further tested for cross-homology by using OliCheck. A large percentage of these probes (83%) were sorted out by OliCheck because they failed specificity criteria defined by Hughes *et al *[15].

### Comparison of *in silico*-predicted with experimentally detected hybridization signals

**Figure 2:**
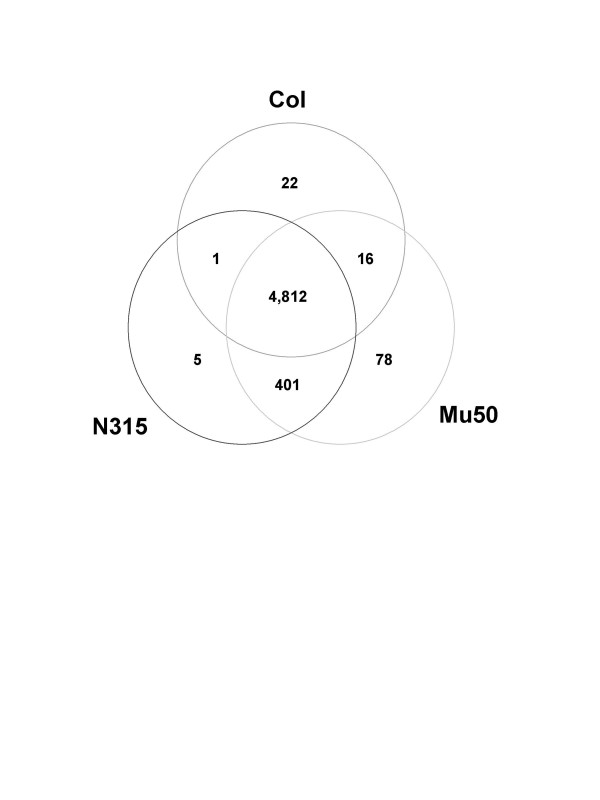
**In silico specificity of selected probes**. Venn diagram showing probes recognized by all strains or strain-specific. Whereas the vast majority of probes recognized target in all three strains (4,812 probes), other recognized only 1 or 2 strains (n = 523).

Among the total number of 5,427 *S. aureus *probes, 4,812 (89%) recognized targets common to all three strains (Fig. 2), thus affording StaphChip valuable properties for comparative genomic and transcriptomic studies on *S. aureus*. The finding of a significant number of probes (n = 401) commonly identified in N315 and Mu50 but not COL may be explained by their closely-related genomic contents [16].

The number of probes predicted to detect genomic elements from each *S. aureus *strain was compared to those experimentally recorded using StaphChip (Table 1). For each strain, >99.5% of the *in silico-*predicted hybridization signals were indeed detected by hybridization of genomic DNA on StaphChip probes. Most of the false-positive signals were recorded on probes that were generated using ArrayDesigner™ only (n = 92/104). *In silico *analysis determined that these false-positive signals did not originate from recognition of intergenic regions (data not shown). Thus, such false-positive frequency is not transferable to the whole array.

**Table 1:** Number of negative and positive signals predicted by *in silico *analysis and recorded by comparative genome hybridization on StaphChip for N315, Mu50, or COL targets. Differences between expected and recorded signals are also shown.

		*S. aureus *genome		
		N315	Mu50	COL

Positive Signal	Predicted	5,219	5,307	4,851
	Recorded	5,216	5,304	4,819
	Missed	3	3	32

Negative Signal	Predicted*	116	28	484
	Recorded	107	20	397
	Differences	9	8	87

*See Figure 2.

### Use of StaphChip for deletion mapping and genomotyping applications

**Figure 3:**
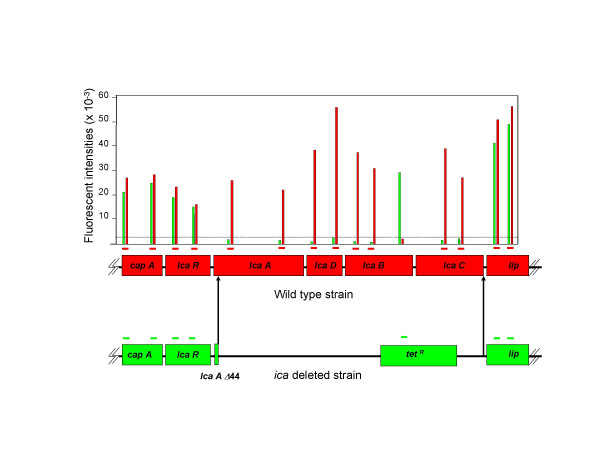
**Mapping of a deleted gene region by StaphChip. **Cy-5 labelled DNA of parental strain SA113 was co-hybridized with Cy-3 labelled DNA from its isogenic *ica *deletion mutant. Colored bars indicate the position of each probe used to map *ica*-related and adjacent ORFs. Background signals (green) were recorded from probes recognizing the *ica-*region known to be deleted in strain SA113*ica *(arrows), as opposed to positive red signals recorded in the wild-type strain. The tetracycline resistance marker used for the construction and selection of strain SA113*ica *is recorded in the green channel only. Dye swap experiments provided similar results (not shown). Data are raw signal intensities; background level is indicated by a dotted line.

To evaluate the accuracy of StaphChip for deletion mapping, the Cy-3 labelled DNA from the SA113*ica *deletion mutant  [20] was competitively hybridized with the Cy-5 labelled DNA from its isogenic parent. Figure 3 maps the *ica*-related signals which are missing in the *ica *mutant, as opposed to the unique positive signal generated by the tetracycline resistance marker used for the construction and selection of strain SA113*ica*. Fluorescent intensities of all other signals except *ica-*related genes were equivalent for both strains.

**Figure 4:**
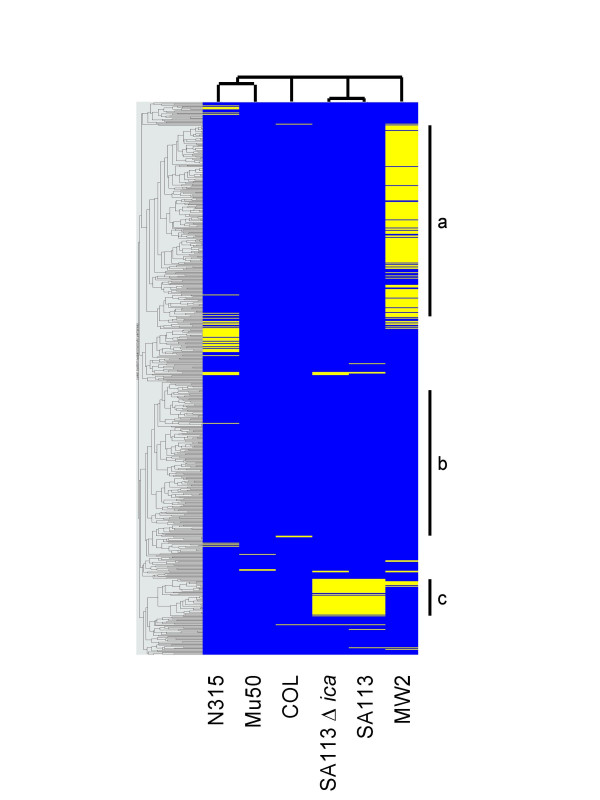
**Comparative genome hybridization using clustering analysis. **Genomic DNA of each individual *S. aureus *strain was labelled with Cy3 and co-hybridized with equivalent amounts of Cy5-labelled genomic DNA pooled from N315, Mu50 and COL. Background-subtracted data were expressed as Log_10 _ratios and analyzed by two-way clustering using GeneSpring 6.1. Probes yielding positive and negative signals are shown in blue and yellow, respectively. The significance of black bars (a, b, and c) is indicated in the text. Note that the figure resolution does not allow visualising single probe differences but only clusters of probes.

The potential of StaphChip for epidemiological typing was analysed by comparative genomic hybridization (CGH) of *S. aureus *strains from various epidemiological origins. Genomic DNA from each individual *S. aureus *strain labelled with Cy3 was co-hybridized with equivalent amounts of Cy5-labelled control genomic DNA (pooled from N315, Mu50 and COL) and analyzed by two-way hierarchical clustering (Fig. 4).

The recently sequenced community-acquired MRSA strain MW2 [17], not included in StaphChip probe design, revealed important differences with strains N315, Mu50, and COL, as shown by a major yellow region on Figure 4. The set of probes yielding negative signals with MW2 DNA (Fig. 4, cluster **a**) corresponds to ORFs coding for antibiotic and heavy metal resistance determinants, bacterial adhesins and DNA modification enzymes lacking in this epidemic strain [17]. In contrast, extensively conserved genomic regions (Fig. 4, cluster **b**) are composed of house-keeping genes contributing to cell-wall, DNA synthesis, as well as essential metabolic enzymes.

Further analysis of strain SA113 and SA113*ica *compared to the other strains, showed no hybridization signals (Fig. 4, cluster **c**) for a number of genes (e.g. exotoxins and antibiotic resistance determinants) known to be missing in pathogenicity islands I-II-III of the NCTC8325 family [21].

### Application of StaphChip for expression profiling studies

Control experiments were performed to study the dose-response of labelled cDNA and influence of hybridization temperature on the linearity and intensities of fluorescent signals. Two portions of N315 cDNA were labelled during reverse-transcription, with either Cy-3 or Cy-5 and competitively hybridized on StaphChip. Characteristics of fluorescent signals obtained on N315 specific probes were compared to those recorded on control *E. coli *oligonucleotide probes, at either 55 or 60°C. The median level of fluorescence intensities were approximately 5–10 fold higher for *S. aureus *probes compared to *E. coli *probes.

On *S. aureus *capture elements, log-transformed signal intensities recorded with equivalent input amounts (5 or 10 μg) of Cy-3 and Cy-5 cDNA were highly correlated (R > 0.95). Linear regression of Cy-3 *versus *Cy-5 scatter plots yielded slopes from 0.94 to 1.02 at 55 or 60°C (Table 2). When 5 μg of Cy-3 was competitively hybridized with 10 μg of Cy-5 labelled cDNA, slopes from 0.48 to 0.53 were recorded at 55 or 60°C, as expected (Table 2). Since log-transformed signal intensities remained highly correlated (R>0.85), this assessed the robustness of the recorded signals. Furthermore, the median intensity of fluorescent signals was marginally altered by temperature changes (not shown).

**Table 2:** Comparison of fluorescent signals obtained with *S. aureus *transcripts hybridized on specific (*S. aureus*) *versus *non-specific (*E. coli*) oligonucleotide probes as a function of target amounts and hybridization temperatures.

	***S. aureus *probes**		***E. coli *probes**	
	R_Cy3/Cy5_	Slope	R_Cy3/Cy5_	Slope

Temp. 55°C Cy3/Cy5: 5 μg/5 μg	0.95	1.02	0.95	1.2
Temp. 55°C Cy3/Cy5: 10 μg/10 μg	0.99	0.94	0.95	1.2
Temp. 55°C Cy3/Cy5: 5 μg/10 μg	0.86	0.48	0.38	0.39
Temp. 60°C Cy3/Cy5: 5 μg/5 μg	0.98	0.94	0.96	1.78
Temp. 60°C Cy3/Cy5: 10 μg/10 μg	0.98	0.94	0.98	1.25
Temp. 60°C Cy3/Cy5: 5 μg/10 μg	0.85	0.53	0.61	0.72

In contrast, signal intensities from *E. coli *control probes were highly disturbed by temperature changes or by altering the ratios of fluorescently-labelled *S. aureus *cDNA (Table 2). Furthermore, the median intensity of fluorescent signals decreased by >2 fold at 60 compared to 55°C (not shown). Dye-swap experiments yielded equivalent results (not shown).

### Reproducibility of StaphChip for expression profiling

**Figure 5:**
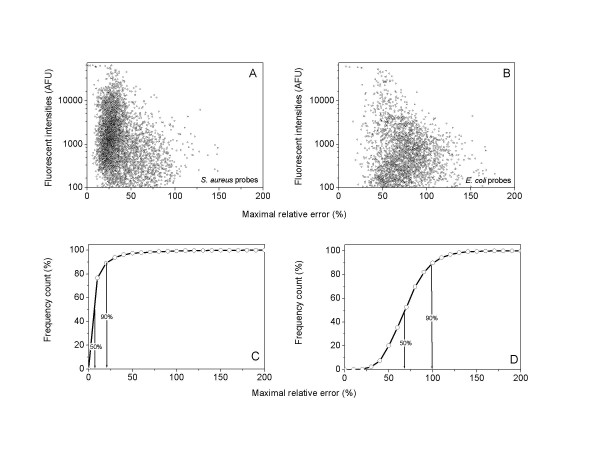
**Reproducibility of fluorescent signals in replicate experiments. **Signals generated on StaphChip by 10 μg Cy-3 labelled N315 cDNA hybridized at 60°C. Average fluorescence intensities from replicate experiments (n = 8) and their maximal relative errors on *S. aureus *(A) or *E. coli *(B) capture probe elements are presented as scatter plots. The cumulative distribution of maximal relative errors is shown for *S. aureus *(C) or *E. coli *(D).

To evaluate the reproducibility of fluorescent signals (Fig. 5), eight independent hybridization experiments were performed under identical conditions using Cy-3 labelled N315 cDNA, derived from overnight cultures. Maximal relative errors [(average-min)/average] of fluorescent probe signal intensities were more widely scattered on *E. coli *(Fig. 5B) compared to *S. aureus *(Fig. 5A) capture elements, thus reflecting the instability of poorly specific interactions with *S. aureus *targets and *E. coli *probes. The 90^th ^percentile of maximal relative errors from *S. aureus *probes represented <25% of average signal intensities. In contrast, the same percentile of maximal relative errors recorded from *E. coli *probes represented >100% of average signal intensities. Furthermore, the 50^th ^percentile on *E. coli *probes was superior to the 90^th ^percentile of maximal relative errors on *S. aureus *probes.

**Figure 6:**
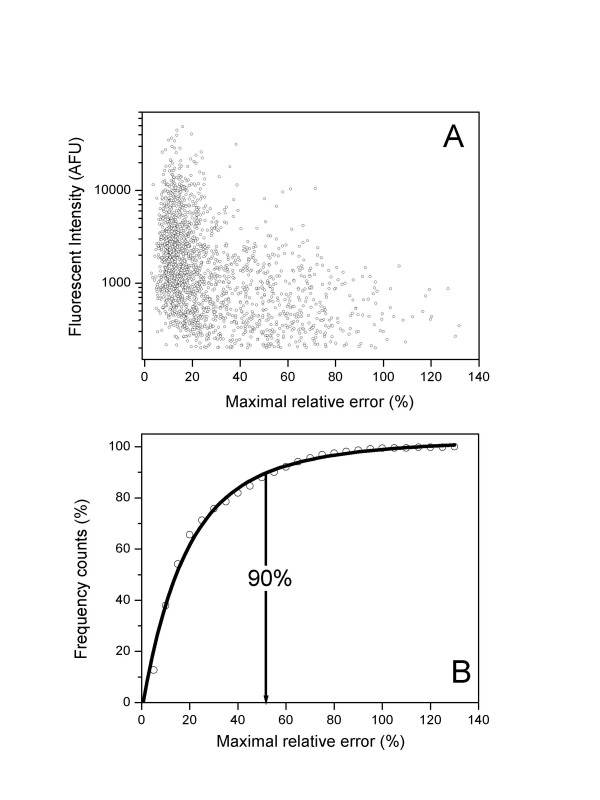
**Reproducibility of fluorescent signals recorded from multiple non-overlapping capture elements for common transcripts. **10 μg Cy-3 labelled N315 cDNA were hybridized at 60°C on StaphChip. For 2,269 selected transcripts detected by two or multiple probes (n = 5,079), average fluorescence intensities and their maximal relative errors are presented in panel (A), and the cumulative distribution of maximal relative errors in panel (B).

Finally, we compared signal intensities generated from individual gene transcripts covered by two or more adjacent but non-overlapping *S. aureus *probes. Figure 6 shows average fluorescence intensities and their maximal relative errors (A) and the cumulative distribution of maximal relative errors (B) For nearly 90% of the gene transcripts (n = 2,269), maximal relative errors of ORF-related signals were <60%. This yields evidence that multiple probes recognizing the same transcripts provide reproducible signals and that StaphChip provides reliable and robust determinations of genome-wide transcripts.

### Validation of relative transcript levels with RT-qPCR

**Figure 7:**
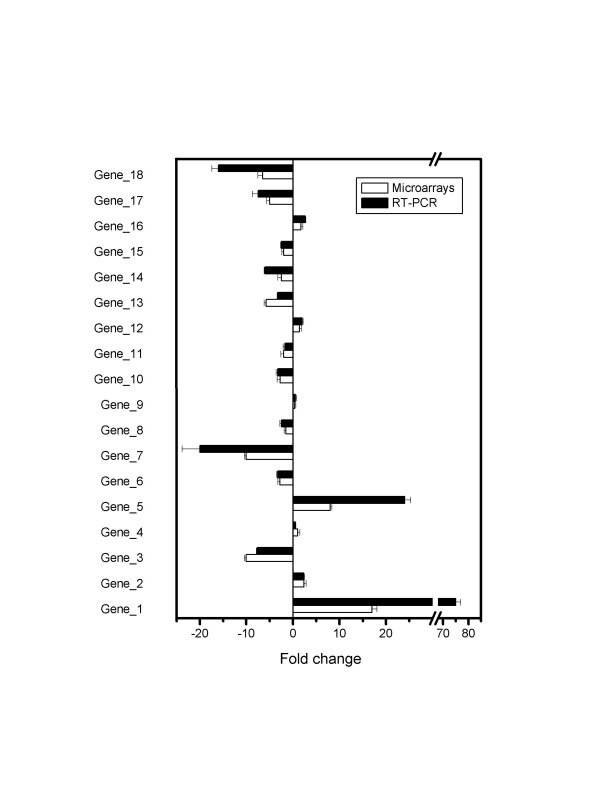
**Comparison of gene expression changes by real-time quantitative PCR and microarray analysis. **Fold changes of gene expression estimated by either technique are shown for a set of 18 genes of *S. aureus *tested in two metabolic conditions. Data represent average values ± standard error of the mean of three independent experiments performed in duplicates.

Figure 7 shows the fold changes estimated by quantitative RT-PCR for 18 transcripts found to be either up regulated, down regulated or unchanged on the StaphChip. A good fitting was observed between both methods, with a correlation coefficient of 0.91 obtained from linear fitting. Of note, the quantitative changes recorded with RT-qPCR tended to be higher than those quantified with the microarray. This finding is supported by previous observations that the dynamic range of RT qPCR is higher than that of microarray [22,23], that only reach 2–3 orders of magnitude. These data validate the use of our method for quantitative gene-expression analysis.

## Discussion

Several recent studies have shown the usefulness of microarrays for genomic and global transcriptomic studies of microbial pathogens [23-25]. Each step of microarray experiments needs to be optimized and validated, from array design and manufacture to data collection and analysis. Among critical technical parameters that need to be controlled are microarray surface chemistry, probe sequence, probe deposition process, and hybridization conditions. Accuracy of microarray-generated data can be improved by using multiple replicates, dye swaps and statistical data analysis. Compared to cDNA microarrays, oligoarrays provide a flexible design and are considered more reliable in terms of sensitivity and specificity [26-29]. Reported drawbacks of cDNA or PCR probes include: i) unpredicted secondary structures, ii) uncontrolled cross-hybridization occurring on repeats or partially homologous regions of PCR probes, and iii) varying amounts and purity of spotted products [27,30]. Recent software development allows genome-wide selection of sub-sequences that uniquely identify genes. Ideally, these approaches should amplify fragments of constant length, thus minimizing the differences in PCR amplification efficiency as well as in hybridization kinetics [31]. However, the extent of cross-hybridization has rarely been evaluated and reported, and thus may lead to severe errors in higher level data analysis, such as clustering [32], genome composition analysis and genotyping for molecular epidemiology [4].

Most oligoarray applications dedicated to prokaryotes were developed by companies using proprietary algorithms [33,34] whose detailed properties are rarely available. Furthermore, the lack of published validation data prevents adequate comparison of those short-probe oligoarrays with investigator-designed oligonucleotide arrays.

To date, several strategies for oligoarray design have been described, but their experimental validation is often limited  [35] or absent [36]. A drawback of these approaches is to apply thermodynamics laws on probe/target interaction as determined in solution [37], but ignoring effects related to oligonucleotide immobilization on a microarray surface [15,38].

To address this issue, OliCheck considered the influence of predicted probe/target binding with respect to its position along the immobilized probe, as demonstrated by Hughes *et al *[15]. This tool allowed selecting probes from large oligonucleotide libraries in order to provide multiple genome coverage, suitable for epidemiological and transcriptomic studies.

As a specific application, OliCheck was used to design StaphChip, an oligoarray dedicated to genomic studies on *S. aureus*, a clinically important pathogen with a low GC content and numerous sequence repeats. The 5,427 feature elements were selected to ideally cover all ORFs of three *S. aureus *genomes with two non-overlapping probes, as validated under experimental conditions. A particular achievement of this strategy is to yield cross-annotations between the designed probes and homologous ORF regions conserved across several genomes. Any new genome sequence can be screened by OliCheck to identify probes that can specifically detect homologous ORFs. Cross-annotations on the recently released *S. aureus *MW2 genome [17] yielded 78% gene coverage. It should be mentioned that the recently published *S. aureus *COL genome [39] composition and annotation have changed significantly since the early release by TIGR in 2003.

The properties of StaphChip design were confirmed by comparative genome hybridization and global gene expression studies. Work in progress assesses the reliability of StaphChip for monitoring *S. aureus *transcription changes during biofilm formation, endocytic stage, or expression of antibiotic resistance. Another achievement (unpublished data) was the design of oligonucleotide probes for the genomes of *Neisseria meningitidis *A and B, *E. coli *K12, *Erwinia carotovora and E. chrysanthemi*; having GC contents ranging from 32.8 to 52%. Furthermore, OliCheck design expands oligoarray use for the study of host-pathogen interactions by potentially preventing cross-hybridization between bacterial probes and contaminating host nucleic acids.

## Conclusion

In summary, this work describes a validated approach to select optimal oligoarray capture elements for *S. aureus *expression analysis and comparative genome hybridization studies. This generic approach will enable researchers to develop customized oligoarrays for genomic studies of any sequenced microorganism.

## Methods

### Design of specific oligonucleotide probes

#### Step A

An initial set of candidate oligonucleotide probes was generated by ArrayDesigner™ 1.17 (Premier Biosoft Intl) using the following probe parameters: (i) 40–60 bp probe length, (ii) 65 ± 10°C target Tm, (iii) <-5.0 kcal/mol for hairpins, (iv) <-8.0 kcal/mol for self-dimers, and (v) dinucleotide repeats shorter than 5 bp (Supplementary material provides OliCheck input format description, for using other probe design software or probe list). The program tested separately each open reading frame (ORF) of the different *S. aureus *genomes freely available at NCBI (*S. aureus *N315 [Genbank# BA000018], Mu50 [Genbank#BA000017]), and TIGR (*S. aureus *COL [TIGR unfinished microbial genome, released in March 27, 2003]). A comprehensive list of all possible probes ranked according to thermodynamic criteria was provided for each genome (Fig. 1a).

Further selection of specific oligonucleotide probes was performed by the design of an original program called OliCheck. This approach is derived from the experimental findings of Hughes *et al*. that microarray hybridization signals are mostly influenced by mismatches in the solution-end (distal part) rather than surface-end (proximal part) portion of the oligonucleotide probe  [15]. OliCheck queries the locally available *S. aureus *genome databases by performing a BLAST (BLASTN for Windows, version 2.2.2) [9] analysis for each probe.

#### Step B

A first probe selection is performed by aligning each probe against its own genome, e.g. N315 candidate probes against N315 genome (Fig. 1b, and 1c).

Each BLAST output is analyzed to extract alignment information. The best scored target-probe alignment (perfect match) is considered as the target of the probe, therefore exempted from further testing. To avoid cross-hybridization, all other alignments are tested using the specificity filtering test. For this test, any 60-mer probe that would align to the target with >11 matched nucleotides by its distal third (solution-end), or >31 matched nucleotides by its proximal two-thirds (surface-end) is rejected. An additional condition is the exclusion of irrelevant target-probe pairs with >18 consecutive nucleotide matches.

#### Step C

Further probe selection is achieved by aligning each candidate probe from each genome (e.g. N315) against the genomes of the other *S. aureus *strains (e.g. Mu50 and COL) (Fig. 1d, and 1e). This process allows annotating probes detecting homologous targets in the other genomes.

Each BLAST output is analyzed to extract alignment information. The best scored target-probe alignment is tested to predict a high hybridization signal. This efficiency test requires the absence of any mismatch in the distal half and <20 mismatches in the proximal half of the probe. If those requirements are not fulfilled, the probe is rejected; otherwise the alignment is considered appropriate for detecting a homologous target and the process is continued. To avoid cross-hybridization with targets from other genomes, further alignments obtained with that probe are checked by the specificity filtering test, as defined in step B. Each probe fulfilling these requirements is annotated as detecting a unique homologous sequence target.

#### Step D

Using a spreadsheet program, the probes were sorted by their ORF target names and their best thermodynamic criteria. Probes showing the best combination of thermodynamic criteria and strain coverage were selected for microarray manufacturing (Fig. 1f). In addition to the selected *S. aureus *probes, OliCheck was used with the same parameters to design 2,873 probes specific for *Escherichia coli *K12 (*E. coli *K12 [Genbank# U00096]).

A compiled version of OliCheck compatible with Windows 2000 or XP and written in the Delphi programming language (Delphi 7, Borland) is freely downloadable for non-profit use at Genomic Research Laboratory website [40].

### *In silico *comparison of our algorithm with ArrayOligoSelector

Three probes set of 60-mer probes with homogenous thermodynamic criteria (Tm = 60°C) were generated using default parameters for N315 genome by: i) ArrayDesigner ii) ArrayOligoSelector for all candidate probes, iii) ArrayOligoSelector for the best candidate probes (one per ORF). The output lists generated by ArrayOligoSelector were reformatted to match OliCheck input file format. The list of probes generated by either software was further processed by OliCheck for cross-homology filtering. The sets of probes selected by each method were further compared for homology using BLAST. Alignment showing E-value <1E-20 were considered as homologous.

### Microarray manufacturing

The StaphChip microarray was manufactured by *in situ *synthesis of 8,455 long oligonucleotide probes (Agilent). It consists of 5,427 *S. aureus *and 2,873 *E. coli *specific probes, together with *A. thaliana *control probes for spiked controls.

### Preparation of the labelled nucleic acids

For comparative genome hybridization, each *S. aureus *strain was grown overnight in 2 ml Mueller-Hinton broth (MHB) and total DNA was extracted and purified using DNeasy columns (QIagen) following manufacturer's instructions. DNA purity and concentration were assayed by spectrophotometer. 2 μg DNA were labelled by the Klenow fragment of DNA polymerase I (BioPrime, Invitrogen) with Cyanine-3 or Cyanine-5 coupled dCTP (NEN) for 2 hours at 37°C, then stopped by the addition of 5 μl 0.5 M EDTA. Labelled DNA was purified on QiaQuick columns (Qiagen).

For gene expression analysis, total RNA was extracted from 2 ml exponential or overnight cultures using the Rneasy kit (Qiagen) as previously described [41]. Batches of 5 or 10 μg of total *S. aureus *RNA were spiked with increasing amounts of different *Arabidopsis thaliana *mRNAs (SpotReport, Strategene), used as external calibrators. The RNA mixture was labelled by Cy-3 dCTP or Cy-5 dCTP, using the SuperScript II (Invitrogen) following manufacturer instructions. Labelled cDNA was then purified onto QiaQuick columns.

### Hybridization and scanning parameters

Unless specified, equivalent amounts of cDNA (or genomic DNA) labelled with Cyanine-3 or Cyanine-5, were diluted in 250 μl Agilent hybridization buffer, and hybridized at a temperature of 60°C for 17 hours in a dedicated hybridization oven (Robbins Scientific). For comparative genome hybridization, genomic DNA from each individual *S. aureus *strain was labelled with Cy3 and co-hybridized with equivalent amounts of Cy5-labelled genomic DNA pooled from N315, Mu50 and COL [42]. Slides were washed, dried under Nitrogen flow and scanned (Agilent) using 100% PMT power for both wavelengths. Data were extracted and processed using Feature Extraction™ software (version 5.0, Agilent).

For gene expression analysis, saturated spots were excluded from subsequent analysis. Local background-subtracted signals were corrected for unequal dye incorporation or unequal load of labelled product. The algorithm consisted of a rank consistency filter and a curve fit using the default LOWESS (locally weighted linear regression) method. Spots showing a reference signal lower than background plus two standard deviations were also excluded from subsequent analyses.

For comparative genome hybridization, local background-subtracted data were expressed as Log_10 _ratios and analyzed by two-way clustering using GeneSpring 6.1 (SiliconGenetics).

### Real time, quantitative PCR analysis

The expression of 18 genes involved in metabolic pathways was quantitatively assayed using by 1 step RT-qPCR using a SDS7700 (Applied Biosystems, Framingham, MA). Primers and probes were identified by scanning each gene sequence using the software Primer Express 2.0(Applied Biosystems). All identified sequences were further aligned on the whole genome of sequenced strains to ensure gene specificity and conservation of the target sequence between strains. Optimal concentration of primers and Taqman probes (labelled with FAM in 3' and coupled to TAMRA in 5' as quencher and purchased from Eurogentec, Seraing, Belgium), determined accordingly to manufacturer's instructions were 200 nM and 100 nM per reaction, respectively. Primers and probes were mixed in Platinum qRT-PCR Thermoscript kit (Invitrogen) with 0.4 ng of total purified RNA. Fold changes were calculated after normalization with the expression level of the 16s rRNA gene as previously described [41].

## Authors' contributions

**YC **performed Olicheck implementation, designed microarray and wrote the manuscript. **BG **performed Olicheck implementation, designed microarray and helped writing the manuscript. **PF **participated in the design of the study, designed experiment protocols, performed microarray analysis. **MB **contributed to design experiment protocols and performed microarray experiments. **AR **has been involved in the CGH project. **PV **has been involved in drafting the article and revising it critically for intellectual content. **WS **has made substantial contributions to conception and design. **JS **initiated the study and helped writing the manuscript. All authors read and approved the final manuscript.

## References

[B1] Yershov G, Barsky V, Belgovskiy A, Kirillov E, Kreindlin E, Ivanov I, Parinov S, Guschin D, Drobishev A, Dubiley S, Mirzabekov A (1996). DNA analysis and diagnostics on oligonucleotide microchips. Proc Natl Acad Sci U S A.

[B2] Dubiley S, Kirillov E, Mirzabekov A (1999). Polymorphism analysis and gene detection by minisequencing on an array of gel-immobilized primers. Nucleic Acids Res.

[B3] Kim CC, Joyce EA, Chan K, Falkow S (2002). Improved analytical methods for microarray-based genome-composition analysis. Genome Biol.

[B4] Lucchini S, Thompson A, Hinton JC (2001). Microarrays for microbiologists. Microbiology.

[B5] Eriksson S, Lucchini S, Thompson A, Rhen M, Hinton JC (2003). Unravelling the biology of macrophage infection by gene expression profiling of intracellular *Salmonella enterica*. Mol Microbiol.

[B6] Staudinger BJ, Oberdoerster MA, Lewis PJ, Rosen H (2002). mRNA expression profiles for *Escherichia coli* ingested by normal and phagocyte oxidase-deficient human neutrophils. J Clin Invest.

[B7] Golub TR, Slonim DK, Tamayo P, Huard C, Gaasenbeek M, Mesirov JP, Coller H, Loh ML, Downing JR, Caligiuri MA, Bloomfield CD, Lander ES (1999). Molecular classification of cancer: class discovery and class prediction by gene expression monitoring.. Science.

[B8] (2004). Array Designer 3.01. http://www premierbiosoft com/dnamicroarray/index html.

[B9] Altschul SF, Gish W, Miller W, Myers EW, Lipman DJ (1990). Basic local alignment search tool. J Mol Biol.

[B10] Talla E, Tekaia F, Brino L, Dujon B (2003). A novel design of whole-genome microarray probes for *Saccharomyces cerevisiae* which minimizes cross-hybridization. BMC Genomics.

[B11] Rouillard JM, Herbert CJ, Zuker M (2002). OligoArray: genome-scale oligonucleotide design for microarrays. Bioinformatics.

[B12] Wright MA, Church GM (2002). An open-source oligomicroarray standard for human and mouse. Nat Biotechnol.

[B13] Bozdech Z, Zhu J, Joachimiak MP, Cohen FE, Pulliam B, DeRisi JL (2003). Expression profiling of the schizont and trophozoite stages of *Plasmodium falciparum* with a long-oligonucleotide microarray. Genome Biol.

[B14] Nielsen HB, Wernersson R, Knudsen S (2003). Design of oligonucleotides for microarrays and perspectives for design of multi-transcriptome arrays. Nucleic Acids Res.

[B15] Hughes TR, Mao M, Jones AR, Burchard J, Marton MJ, Shannon KW, Ziman M, Meyer MR, Kobayashi S, Dai H, He YD, Stephaniants SB, Cavet G, Walker WL, West A, Coffey E, Shoemaker DD, Stoughton R, Blanchard AP, Friend SH, Linsley PS (2001). Expression profiling using microarrays fabricated by an ink-jet oligonucleotide synthesizer. Nat Biotechnol.

[B16] Kuroda M, Ohta T, Uchiyama I, Baba T, Yuzawa H, Kobayashi I, Cui L, Oguchi A, Aoki K, Nagai Y, Lian J, Ito T, Kanamori M, Matsumaru H, Maruyama A, Murakami H, Hosoyama A, Mizutani-Ui Y, Takahashi NK, Sawano T, Inoue R, Kaito C, Sekimizu K, Hirakawa H, Kuhara S, Goto S, Yabuzaki J, Kanehisa M, Yamashita A, Oshima K, Furuya K, Yoshino C, Shiba T, Hattori M, Ogasawara N, Hayashi H, Hiramatsu K (2001). Whole genome sequencing of meticillin-resistant *Staphylococcus aureus*. Lancet.

[B17] Baba T, Takeuchi F, Kuroda M, Yuzawa H, Aoki K, Oguchi A, Nagai Y, Iwama N, Asano K, Naimi T, Kuroda H, Cui L, Yamamoto K, Hiramatsu K (2002). Genome and virulence determinants of high virulence community-acquired MRSA. Lancet.

[B18] Gill SR (2004). *Staphylococcus aureus* COL genome. http://www tigr org/tigr-scripts/CMR2/GenomePage3 spl?database=gsa.

[B19] Blattner FR, Plunkett GIII, Bloch CA, Perna NT, Burland V, Riley M, Collado-Vides J, Glasner JD, Rode CK, Mayhew GF, Gregor J, Davis NW, Kirkpatrick HA, Goeden MA, Rose DJ, Mau B, Shao Y (1997). The complete genome sequence of *Escherichia coli* K-12. Science.

[B20] Cramton SE, Gerke C, Schnell NF, Nichols WW, Gotz F (1999). The intercellular adhesion *(ica)* locus is present in *Staphylococcus aureus* and is required for biofilm formation. Infect Immun.

[B21] Novick RP, Schlievert P, Ruzin A (2001). Pathogenicity and resistance islands of staphylococci. Microbes Infect.

[B22] Ramakrishnan R, Dorris D, Lublinsky A, Nguyen A, Domanus M, Prokhorova A, Gieser L, Touma E, Lockner R, Tata M, Zhu X, Patterson M, Shippy R, Sendera TJ, Mazumder A (2002). An assessment of Motorola CodeLink microarray performance for gene expression profiling applications. Nucleic Acids Res.

[B23] Nuwaysir EF, Huang W, Albert TJ, Singh J, Nuwaysir K, Pitas A, Richmond T, Gorski T, Berg JP, Ballin J, McCormick M, Norton J, Pollock T, Sumwalt T, Butcher L, Porter D, Molla M, Hall C, Blattner F, Sussman MR, Wallace RL, Cerrina F, Green RD (2002). Gene expression analysis using oligonucleotide arrays produced by maskless photolithography. Genome Res.

[B24] Wang D, Coscoy L, Zylberberg M, Avila PC, Boushey HA, Ganem D, DeRisi JL (2002). Microarray-based detection and genotyping of viral pathogens. Proc Natl Acad Sci U S A.

[B25] Okamoto T, Suzuki T, Yamamoto N (2000). Microarray fabrication with covalent attachment of DNA using bubble jet technology. Nat Biotechnol.

[B26] Blanchard AP, Friend SH (1999). Cheap DNA arrays-it's not all smoke and mirrors. Nat Biotechnol.

[B27] Barczak A, Rodriguez MW, Hanspers K, Koth LL, Tai YC, Bolstad BM, Speed TP, Erle DJ (2003). Spotted long oligonucleotide arrays for human gene expression analysis. Genome Res.

[B28] Kothapalli R, Yoder SJ, Mane S, Loughran TPJ (2002). Microarray results: how accurate are they?. BMC Bioinformatics.

[B29] Li J, Pankratz M, Johnson JA (2002). Differential gene expression patterns revealed by oligonucleotide versus long cDNA arrays. Toxicol Sci.

[B30] Yang IV, Chen E, Hasseman JP, Liang W, Frank BC, Wang S, Sharov V, Saeed AI, White J, Li J, Lee NH, Yeatman TJ, Quackenbush J (2002). Within the fold: assessing differential expression measures and reproducibility in microarray assays. Genome Biol.

[B31] Haas SA, Hild M, Wright AP, Hain T, Talibi D, Vingron M (2003). Genome-scale design of PCR primers and long oligomers for DNA microarrays. Nucleic Acids Res.

[B32] Quackenbush J (2002). Microarray data normalization and transformation. Nat Genet.

[B33] Rosenow C, Saxena RM, Durst M, Gingeras TR (2001). Prokaryotic RNA preparation methods useful for high density array analysis: comparison of two approaches. Nucleic Acids Research.

[B34] Dunman PM, Murphy E, Hanney S, Palacio D, Tucker-Kellogg G, Wu S, Brown EL, Zagurski RJ, Shlaes D, Projan SJ (2001). Transcription Profiling-Based Identification of *Staphylococcus aureus* Genes Regulated by the *agr* and/or *sarA* Loci. J Bacteriol.

[B35] Tolstrup N, Nielsen PS, Kolberg JG, Frankel AM, Vissing H, Kauppinen S (2003). OligoDesign: Optimal design of LNA (locked nucleic acid) oligonucleotide capture probes for gene expression profiling. Nucleic Acids Res.

[B36] Rouillard JM, Zuker M, Gulari E (2003). OligoArray 2.0: design of oligonucleotide probes for DNA microarrays using a thermodynamic approach. Nucleic Acids Res.

[B37] SantaLucia JJ (1998). A unified view of polymer, dumbbell, and oligonucleotide DNA nearest-neighbor thermodynamics.. Proc Natl Acad Sci U S A.

[B38] Ketomaki K, Hakala H, Kuronen O, Lonnberg H (2003). Hybridization properties of support-bound oligonucleotides: the effect of the site of immobilization on the stability and selectivity of duplex formation. Bioconjug Chem.

[B39] Gill SR, Fouts DE, Archer GL, Mongodin EF, Deboy RT, Ravel J, Paulsen IT, Kolonay JF, Brinkac L, Beanan M, Dodson RJ, Daugherty SC, Madupu R, Angiuoli SV, Durkin AS, Haft DH, Vamathevan J, Khouri H, Utterback T, Lee C, Dimitrov G, Jiang L, Qin H, Weidman J, Tran K, Kang K, Hance IR, Nelson KE, Fraser CM (2005). Insights on evolution of virulence and resistance from the complete genome analysis of an early methicillin-resistant *Staphylococcus aureus* strain and a biofilm-producing methicillin-resistant *Staphylococcus epidermidis* strain. J Bacteriol.

[B40] (2004). Genomic Research Laboratory. http://www genomic ch/software php.

[B41] Vaudaux P, Francois P, Bisognano C, Kelley WL, Lew DP, Schrenzel J, Proctor RA, McNamara PJ, Peters G, von Eiff C (2002). Increased expression of clumping factor and fibronectin-binding proteins by *hemB* mutants of *Staphylococcus aureus* expressing small colony variant phenotypes. Infect Immun.

[B42] Talaat AM, Howard ST, Hale W, Lyons R, Garner H, Johnston SA (2002). Genomic DNA standards for gene expression profiling in *Mycobacterium tuberculosis*. Nucleic Acids Res.

